# Exploring Key Factors Driving Urban Foraging Behavior in Garden and Non-Garden Locations

**DOI:** 10.3390/foods12051032

**Published:** 2023-02-28

**Authors:** Meike Rombach, David L. Dean

**Affiliations:** 1 Department of Land Management and Systems, Lincoln University, Lincoln 7647, New Zealand; 2 Department of Agribusiness and Markets, Lincoln University, Lincoln 7647, New Zealand

**Keywords:** alternative food procurement, foraging attitudes, urban foraging

## Abstract

Since the occurrence of COVID-19 and food price inflation, alternative forms of food procurement increased in popularity. The present study is dedicated to urban foraging and aims to explore key factors driving food foraging behavior in the U.S. Two specific foraging behaviors, namely “leaving food behind” or “taking it all”, have been investigated in a gardening and non-gardening location. Leaving food behind is crucial to sustainable foraging practices, as it allows plants and ecosystems to recover and promotes fairness in foraging communities. Data was procured from an online consumer survey and analyzed using SmartPLS 4, which allowed the use of partial least square structural equation modeling (PLS-SEM). PLS-SEM is particularly suitable for complex exploratory studies as it does not require distributional assumptions. Results indicate that nature and food attitudes predict attitudes toward urban foraging. Foraging attitudes, such as food foraging is challenging and food foraging benefits people and the planet, which are the most important drivers for taking or leaving behaviors in both types of locations. These findings are of relevance to managers in municipalities, landscape designers, horticultural businesses, and other stakeholders who create, shape, and govern landscapes used for food foraging.

## 1. Introduction

For the past decade, alternative means of food consumption and procurement have been increasing in popularity in the U.S. [1,2]. These correspond with rises in urban horticulture, green cities, and the establishment of informal initiatives and formal programs to build community gardens or plant trees [3,4,5,6]. Since the occurrence of COVID-19, the trend has broadened to include home gardening, do-it-yourself, and food foraging, which some attribute to regional recessions and food price inflation in the U.S. [7,8,9,10]. Food foraging is a consumer behavior that refers to the self-provisioning of plants found in rural and urban landscapes that are suitable for human consumption [11]. It requires searching, identifying, and collecting wild edible plants, such as fruits, nuts, mushrooms, herbs, and roots [12,13,14]. Evidence for the increase in popularity of food foraging includes foraging tours and online and face-to-face classes offered by educational providers in the U.S., with notable offerings from Washington College and Masterclass [15,16]. Less formal examples include the California-based “ForageFS”, which offers tours to learn how to harvest mushrooms, wild plants, and seaweed [17]. Similarly, the “Beacon Food Forest” in Seattle offers environmental education and foraging events. In addition, there are private and governmental web pages that provide advice on foraging locations and practices [18]. For instance, the project “Falling Fruit” displays locations around the US where food foraging is permitted. Small dots pinpoint these foraging locations and provide species information and suggest the appropriate windows to harvest fruit. These information sources provide links to the U.S. Department of Agriculture’s (USDA) homepage for further information [19]. Furthermore, food foraging is discussed and organized in social media groups and apps [9].

Food foraging allows U.S. consumers to mitigate some food insecurity while feeling a closer connection to nature [20,21]. Both aspects are beneficial as COVID-19 brought hardship to many communities, and in its initial stages posed a risk to physical and mental well-being [22,23]. Since the beginning of the pandemic, well-being, unemployment, reduced available household incomes, and food insecurity were reasons that generated people’s interest in home food procurement, including food foraging [24,25,26,27]. Data from the Bureau of Labor Statistics indicates higher unemployment rates from 2020 to 2021 than before the pandemic, and a household survey conducted by the United States Census Bureau reported that 48% of the respondents felt either depressed or anxious. Concerning food insecurity, the Economic Research Service of the USDA emphasizes that over 10% of households were food insecure in 2020 and 2021 [25,26,27]. Lockdowns and physical distance limitations led to a decrease in exposure to nature and a reduction in well-being. For many U.S. citizens, contact with nature reduces stress and improves their quality of life [28,29].

Despite these benefits, some U.S. cities do not permit food foraging and consider it to be illegal [30,31,32,33,34]. Urban foraging is forbidden in traditional conservation areas, as the activity could threaten specific species or the overall ecosystem stability [22]. Webpages dedicated to individual states, such as “Foraging Texas” outline the legal situation, ethical foraging behavior, and plant species being permitted or forbidden to harvest across locations and times of the year [35]. The best practice recommendation on these web pages aligns with findings from the extant literature [35]. Following Ticktin (2004) and Schunko et al. (2021), food foraging may negatively impact individual plant species’ ability to grow and reproduce. This could result in adverse effects on plant populations, plant communities, and on the overall ecosystem [22,36].

In addition, food foraging can be discouraged, if not actively restricted, in intensively managed parks and greenspaces [9,22]. The restrictions often allow municipalities to perform maintenance work undisturbed and avoid vandalism, dog exposure, injury, or disrespect towards sites with specific purposes, such as botanic gardens or graveyards [9,36]. In terms of location and food foraging practice, the recent body of literature addresses best practice recommendations for food foraging. These acknowledge the needs of foragers, ecosystems, and legal frameworks [11,16].

Consequently, food foragers should be knowledgeable of plant species and responsible selection practices [37,38]. Various studies emphasize the importance of carefully selecting species and locations [14,39]. It is considered irresponsible to forage plants susceptible to harvesting pressure or those under protection [39]. Instead, responsible foraging practices include foraging in multiple locations to allow plants to regrow and conscious harvesting to avoid damaging branches or root systems. Overall, plant knowledge and sustainable practice considering ecosystems and other foragers is required [9,11,16,37,39]. Schunko et al. (2021) discuss seasonal-appropriate foraging times and practices that are considered as best practices [16]. For example, foragers should avoid young plants, which are unripe, and avoid any practice that disturbs maintenance activities in public greenspace [16,39].

While these practices and food-foraging behavior have been investigated as a social phenomenon, various people and plant disciplines contribute to the body of literature. Anthropological, sociological, ecological, agricultural, and forestry studies are common [12,16,34,40,41,42], but studies dedicated to consumer behavior are relatively scant. The recent body of literature indicates that food foraging takes place in various locations. While Shackleton et al. (2017) indicate that foraging takes place in rural, urban, and semi-urban areas [34], Landor Yamagata et al. (2018) outline private and public locations, for instance forests, allotment gardens, cemeteries, campuses, sports fields, and roadsides [20]. Building on these studies, Fisher and Kowarik (2020) and Brandner and Schunko (2022) critically discuss that foraging behavior is varying within these locations. Brandner and Schunko (2022) emphasize that specific foraging behavior across locations and factors driving foraging behavior are yet to be explored in more detail [42,43]. So far, only spatial factors influencing access to foraging behavior have been explored. Hence, this study aims to fill this research gap and contribute a new perspective by focusing on attitudinal and perceptional factors as drivers of foraging behavior. The present study examines critical factors driving U.S. consumers’ food foraging behavior in varying locations. More precisely, the study focuses on the garden and non-garden locations with varying degrees of perceived appropriateness and looks specifically into whether U.S. foragers follow best practice recommendations and forage everything available or leave plants behind as an indication of their respect for nature and other foragers.

## 2. Background of the Study

**Figure 1:**
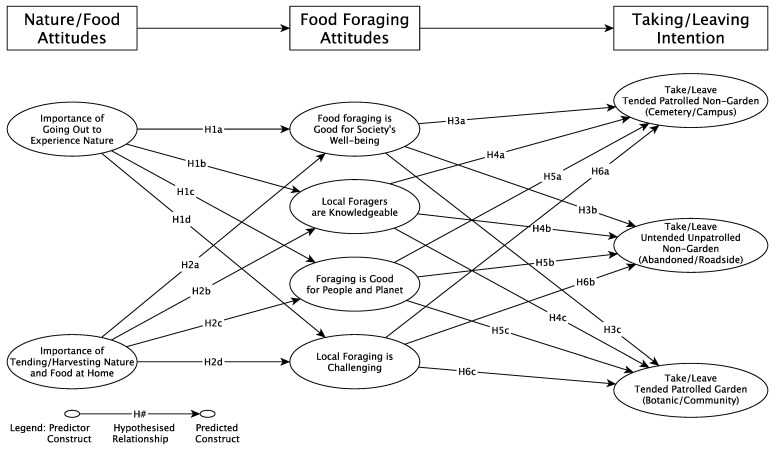
Conceptual Model of Food Foraging Behavior (Taking/Leaving).

This section comprises a literature review supporting the conceptual model and the corresponding hypotheses (see Figure 1). It also includes a description of the survey instrument, the sampling approach, as well as information about data collection and analysis.

### 2.1. Nature Experience

Previous studies indicate that food foragers usually have pro-social and environmental attitudes [32,44,45] and consider nature experiences essential to their lifestyle and identity [16]. Nature is often experienced through food-sourcing-related activities, such as hunting and fishing, or recreational activities, such as camping, hiking, sailing, surfing, and boating [12,21,36,41]. For individuals involved in foraging, sourcing food items and natural materials, such as wood or shells, are equally important as the nature experience itself [41,46]. This is because such experiences allow them to be in contact with nature and feel emotionally and spiritually connected [21]. The importance dedicated to outdoor activities and nature-relatedness is often instilled through family and childhood experiences or religion and value systems [11,12,13,14,43,44]. Moreover, as consumers, these individuals consider the effects of their consumption choices on the environment and society [11,43]. Due to the foraging activities and the strong connection between foragers and nature, foragers tend to have good knowledge about plants, ecosystems, the impact of foraging practices on nature as a whole, specific locations, and matters of legality [11,16,40]. Amidst this background, the following multi-part hypothesis is proposed:

**Hypothesis 1 (H1).**  *The importance that consumers dedicate to going out to experience nature positively impact consumers’ food foraging attitudes and perception, such as (a) food foraging benefits societal wellbeing, (b) food foraging benefits people and the planet, (c) local foragers are knowledgeable, and (d) local food foraging is challenging*.

### 2.2. Importance of Tending/Harvesting Nature and Food at Home

The recent body of literature emphasizes a close connection between food foraging activities, such as growing plants, gardening, food production, food processing, as well as animal husbandry, as these activities allow for nature experiences, understanding of food production, the processes and resources required to obtain food, as well as self-sufficiency [13,22]. In U.S. and European metropoles, books, and classes are offered on identifying and cooking with wild plants [9,39], which aim to increase the popularity of food foraging [39] and foster connections between home-based and outdoor activities. Further studies emphasize the importance of complementary home-based/foraging activities to mitigate food insecurity and as a means to share knowledge and skills relevant to culture, ethnicity, or religion [38]. As both types of activities impact foragers’ attitudes and perceptions of the impact of their actions, the following muti-part hypothesis is proposed:

**Hypothesis 2 (H2).**  *The importance that consumers dedicate to tending/harvesting nature and food at home positively impact consumers’ food foraging attitudes and perception such as (a) food foraging benefits societal wellbeing, (b) food foraging benefits people and the planet, (c) local foragers are knowledgeable, and (d) local food foraging is challenging*.

### 2.3. Foraging Is Good for Society’s Well-Being, People, and Planet

Previous studies have found that food foraging contributes to society’s social well-being and development [39]. Good practices among foragers require solidarity and redistributive justice since it is expected to leave food for other foragers [11,16]. These values and practices have become even more critical since the occurrence of COVID-19, as various papers outline foraging as a means to mitigate hardship and food insecurity [9,10]. In this context, Prost et al. (2019) describe food foraging as a participatory activity contributing to an active local food democracy. The authors emphasize the importance of relationships and landscape development [45]. Various studies characterize food foraging as an ethical and sustainable activity [22,28] arguing from social and economic perspectives. However, ecological studies criticize competitive foraging and selection pressures as drivers of unsustainable behavior [28]. Plant knowledge, food preparation knowledge, good foraging practices, and gentle eco-tourism are crucial to environmental sustainability in a foraging context [11,22]. Thus, the following hypotheses are proposed:

**Hypothesis 3 (H3).**  *The importance that consumers dedicate to foraging as an activity that serves the good of societies’ well-being impacts (a) consumers’ taking or leaving intention in tended/patrolled non-garden locations, (b) consumers’ taking or leaving intention in untended/unpatrolled non-garden locations, and (c) consumers’ taking or leaving intention in tended/patrolled garden locations*.

**Hypothesis 4 (H4).**  *The importance that consumers dedicate to foraging as an activity that serves the good of people and the planet impacts (a) consumers’ taking or leaving intention in tended/patrolled non-garden locations, (b) consumers’ taking or leaving intention in untended/unpatrolled non-garden locations, and (c) consumers’ taking or leaving intention in tended/patrolled garden locations*.

### 2.4. Foraging Knowledge and Local Foraging

Various studies have focused on food foraging knowledge having spoken with consumers in rural and urban areas and municipality officials [11,16,45,46]. These studies emphasized foraging quantities, accessibility of locations, tools, dates, and knowledge of local plants and regulations, but their findings have been divergent [47,48,49,50]. For instance, Garekae and Shackleton (2021) dedicated their work to formal and informal regulations governing food foraging [51]. Their study uncovered that the majority of their survey respondents needed to be more knowledgeable of formal and informal rules governing food foraging and the use of urban landscapes [51].

Similarly, Sardeshpande and Shackleton (2020) reported on the experiences of municipal officials and their stakeholder engagement with food foragers [32]. A general lack of knowledge of wild plants, indigenous species, unsustainable foraging, and toxic soils was considered a reason to discourage foraging in urban areas [32]. Both studies recommend policy, ecological and botanic education, and stakeholder management to improve sustainable foraging knowledge and local foraging practices [32,45].

**Hypothesis 5 (H5).**  *The importance that consumers dedicate to foraging knowledge impact (a) consumers’ taking or leaving intention in tended/patrolled non-garden locations, (b) consumers’ taking or leaving intention in untended/unpatrolled non-garden locations, and (c) consumers’ taking or leaving intention in tended/patrolled garden locations*.

**Hypothesis 6 (H6).**  *The importance that consumers dedicate to foraging as challenging local activity impact (a) consumers’ taking or leaving intention in tended/patrolled non-garden locations, (b) consumers’ taking or leaving intention in untended/unpatrolled non-garden locations, and (c) consumers’ taking or leaving intention in tended/patrolled garden locations*.

### 2.5. Survey Instrument and Sampling Approach

The current study uses 34 items from a more extensive 109-item food foraging online survey. The 34 items cover socio-demographic information, attitudes toward nature, food, food foraging, and foraging intention. The data collection utilized Amazon Mechanical Turk (MTurk) in 2022. MTurk is a longstanding crowdsourcing platform used to collect social science data for the last decade [52,53,54]. Respondents were screened to include those who were 18 years or older, reside in the U.S., and have some interest and experience with food foraging. A total of 417 responses were returned, and after the data were cleaned, 401 responses were suitable for the Partial Least Square Structural Equation Modeling (PLS-SEM). The ten-times rule was applied to confirm minimum sample size was achieved [55]. The questionnaire items/scales were drawn from the literature. Questions related to going out to experience nature were adapted from Schunko and Brandner (2022) [11]. Questions related to the importance of tending/harvesting nature and food at home were adapted from Fischer and Kowarik (2020) and Byrd and Widmar (2015) [43,56]. Food foraging attitudes were developed by Sardeshpande and Shackleton (2020) and Schunko and Brandner (2022) [11,32]. All these attitudinal questions used seven-point Likert-type agreement scales (1 = strongly disagree to 7 = strongly agree). Foraging intentions for a variety of locations were developed by the authors and used a 100-point scale anchored by 0 = leave everything to 100 = take everything.

### 2.6. Analysis

A demographic sample description was generated using SPSS, and the PLS-SEM analysis was performed using SmartPLS in two stages. First the outer model was examined (measurement model assessment), then the inner model (structural model assessment). PLS-SEM is appropriate because it is suitable for the examination of complex theoretical models and can accommodate both multi-item and single measures, smaller samples, and relaxed distributional assumptions, especially when compared with covariance-based approaches to SEM [57,58].

PLS-SEM first analyzes the outer model, or the relationships between questions and proposed scales (latent variables), then it tests the inner model identifying the significant relationships between latent variables. The outer model tests the factor loadings of questions to their respective scales. Loadings are recommended to be above 0.4 [48]. Item/scale convergence is tested and considered acceptable when the average variance extracted (AVE) is greater than 0.5 [58]. Scale reliability is measured using traditional Chronbach’s alphas and the more recent composite reliability and is acceptable if greater than 0.6 [58,59]. Discriminant validity is verified in two ways: using the traditional Fornell–Larcker criterion greater than the cross-loadings (Fornell and Larcker, 1981) and the more recent heterotrait–monotrait ratio of correlations criterion (HTMT) [60]. HTMT values should be less than 0.9 and HTMT is a preferred method to test discriminant validity [61,62]. Lastly, multicollinearity is examined via inner model variance inflation factor (VIF) scores and are considered satisfactory if less than 5 [52,53].

The inner model examinations test the structural fit of the model and the model’s explanatory power and predictive relevance. Reporting goodness of fit (GoF) and Normed Fit Indices (NFI) for PLS-SEM models are expected; however, despite higher scores being better, their interpretation is unclear as cautioned by Hair et al. (2022) [57]. Smaller Standardized Root Mean Square Residual (SRMR) indicates a better fit, and the convention is that SRMR values are acceptable if under 0.08 and problematic if over 0.10 [57]. Finally, the model’s explanatory power (R^2^) is considered weak, moderate, or substantial if they are near 0.25, 0.50, and 0.75, respectively. Also, the model’s predictive relevance (Stone–Geisser criterion Q^2^) are considered to have acceptable, medium, and strong predictive relevance if they are greater than 0, 0.25, and 0.50, respectively [57]. Once the inner and outer model analysis criteria are satisfied, the proposed hypotheses are tested. The testing of hypotheses involves examining the direction and statistical significance of the coefficients representing each hypothesized relationship using the PLS-SEM bootstrapping procedures (5000 samples). In many research topics, there is sufficient evidence to hypothesize the positive or negative sign of relationships, but in the absence of such evidence, the hypotheses of the current study do not commit to a sign.

To examine the invariance of the model across subsamples, hierarchical clusters (Ward’s method) were computed based on the foraging intention questions across all the potential locations. The resultant clusters followed tendencies to harvest heavily, which were named “Takers.” Those who tended to balance harvesting and not harvesting were named “balancers”, and those who tended toward little or no harvesting were named “leavers.” While no hypotheses were offered for these clusters, they represent a useful classification and indicate whether the hypotheses were supported across them.

## 3. Results and Discussion

The overall sample description, sub-sample statistics, and relevent US census statistics are display in Table 1. In the overall sample, the percentage of men and women were 50.4% and 49.6%, respectively. Residents from the Southern (51.6%) and Northeastern (17.0%) regions were over-represented relative to U.S. Census data, and residents from the Midwest (16.0%) and Western (6.2%) region were under-represented. Respondents between 25 and 34 years old (51.1%) were the largest age group, and many of the age groups (25–34, 35–44, and 45–54) were over-represented relative to census statistics. Conversely, the young (18–24) and elderly (55–64 and 65+) were under-represented. Additionally, the respondents were better educated but had lower incomes than census statistics. In other U.S. food foraging studies, there is no consensus concerning the influence of socio-demographic background [63,64,65,66]. Participating in food foraging does not seem to be explicitly tied to consumers with specific income levels, but culture and family traditions are important influences. Hence, there is diversity among urban foragers.

Table 2 presents the results from the inner model analysis. Items sufficiently contributed to their respective scales with 0.4 or better factor loadings. Reliabilty criteria was satisfied with all scores above 0.6, and convergent validity was confirmed with all AVE scores above 0.5.

Table 3 shows that discriminant validity was confirmed. Specifically, the Fornell–Larcker criterion was satisfied as the square root of the scale’s AVE was greater than the cross-loadings. HTMT was also satisfied as the HTMT ratios were less than 0.90. Finally, multicollinearity was not an issue in the data set as demonstrated by the VIF values (max: 2.09 and average: 1.79). Thus, all the requirements for testing the measurement (outer) model have been satisfied and the model is deemed suitable for model structure testing.

**Figure 2:**
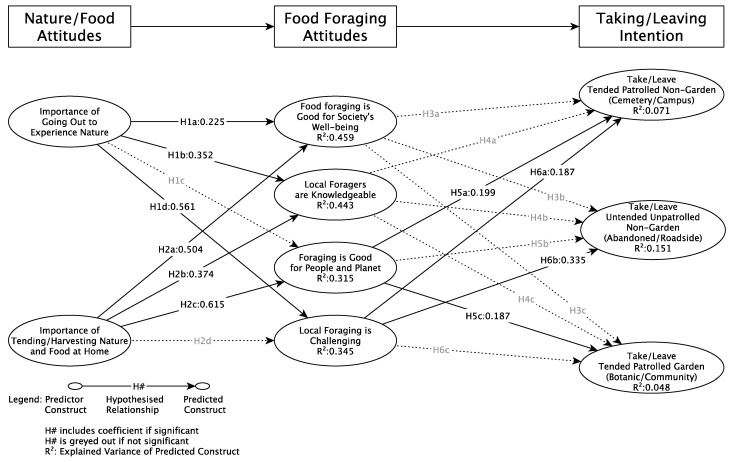
Conceptual Model Results.

Model structure analyses indicate a goodness of fit of 0.426, a normed fit index of 0.701, and an SRMS of 0.068, indicating adequate model fit. The model has acceptable explanatory power and acceptable predictive relevance with average R^2^/Q^2^ values of 0.262/0.229. Figure 2 shows strong explained variance for two of the seven dependent variables as they were near 50% (R^2^:0.5), and two were near 25% (R^2^:0.25) indicating moderate explanatory power.

Figure 2 and Table 4 display the result of the hypothesis testing. In general, there was strong support for the hypothesized relationships between the nature/food attitudes and food foraging attitudes but limited support for the influence of food foraging attitudes on taking/leaving intentions. These findings are well in line with previous studies. Nature attitudes and nature contact are closely associated with food-foraging attitudes [47]. However, their translation into behavior cannot be confirmed by previous studies [11]. Hypothesis 1 was not fully supported as the importance of going out to experience nature significantly influenced three of the four food foraging attitudes: goodness for society (H1a), knowledgeability of foragers (H1c), and challenges of foraging (H1d), but was not a driver of goodness to people and planet (H1b). These findings corroborate with Schunko and Brander (2022) and Sardeshpande and Shackleton (2020) who indicate that food foraging is still frowned upon. Social acceptance is hampered by incidences of unsustainable foraging practices with no concern for ecological systems and other societal obstacles towards food foraging [11,32], especially those that oppose what is seemingly good for people and the planet. Nature experiences, knowledge, and food foraging as means to counteract hardship and justice in food access have been highlighted by previous studies. Hypothesis 2 also did not receive full support as the importance of tending/harvesting nature and food at home only influenced three of the four food-foraging attitudes: foraging goodness for society (H2a), knowledgeability of foragers (H2c), and goodness for people and planet (H2b), but did not influence challenges of foraging. The importance of tending/harvesting food at home and in gardening is emphasized by Fischer and Kowarik (2020). However, associations with attitudes were not covered in their work [43]. The non-significant finding related to the challenges that are associated with food foraging diverge from Fischer and Kowark [43] and Schunko and Brandner (2022) [11]

There was no support for H3 or H4 as neither goodness for society nor knowledgeability of foragers influenced the taking/leaving intention for any of the foraging locations. Some support was found for H5 as good for people and the planet influenced intention towards more harvesting for two of the three locations: tended/patrolled non-garden (H5a) and tended/patrolled garden (H5c). H6 also found partial support as an increased challenge of foraging influenced intention towards more harvesting for both tended/patrolled (H6a) and untended/unpatrolled (H6b) non-garden settings.

When examining the relationships across sub-groups, many of the hypothesized relationships found in the overall sample were also found in the sub-groups (Table 4 with green highlight). These relationships could be considered relatively consistent across the sub-groups. Some relationships were significant in the overall sample but not significant for the sub-samples (yellow highlight). For some, this could have been due to the smaller sample size of the sub-groups. The most dynamic were those that were significant in subsamples but were not in the overall sample (red highlight). For example, foraging goodness for people and the planet seemed to be a driver of intention to harvest in untended/unpatrolled non-gardens for takers but an inhibitor to harvesting for leavers. The reasoning for these behavioral differences is yet to be explored but may be explained by plant and ecological knowledge, knowledge of laws and regulations, as well respect for the common good and resources.

## 4. Conclusions

The study aimed to explore the taking or leaving behavior of food foragers in tended and untended garden and non-garden settings. It was found that nature and food attitudes predict foraging attitudes relatively well. Foraging attitudes, such as food foraging is challenging and food foraging benefits people and the planet, are the most important drivers for taking or leaving behavior. The results of the present study are a valuable addition to the recent body of literature on urban food foraging and are of relevance to municipalities and consumers alike. The foraging attitude “Foraging is good for people and planet” as a predictor for taking and leaving behavior in tended/patrolled garden settings is relevant information for municipalities. Given that foragers are willing to expose themselves to a potential risk of punishment or even litigation in both patrolled settings suggests that municipalities must make permitted foraging spaces, laws, and regulations more transparent. In collaboration with the Natural Resources Conservation Service of the USDA, this can be achieved by improving individual state websites presenting maps with areas where foraging is permitted and not permitted to centralize information. The involvement of a governmental body would increase the trustworthiness and credibility of foraging information compared to the currently existing web pages. In addition, information related to species and soil conditions, for instance, the indication of soil toxicity or protected species, would be helpful to present through virtual reality and educational foraging videos. This would make the information more accessible and attractive to a larger audience interested in food foraging.

For patrolled places, non-garden places, such as campuses, permitting food foraging in dedicated areas may be an opportunity for student–campus engagement and avoiding food losses. Hands-on foraging activities may be an interesting way to combine botany, plant science, plant identification, or culinary studies.

Finally, the data procurement and sampling methods of the present need to be critically reflected upon as the data was obtained from MTurk, a crowdsourcing platform. MTurk samples are not comparable with representative samples of the U.S. population but are superior to convenience samples. However, given that there is so far no consensus on who the food foragers are and their socio-demographic background profiles in the U.S., a diverse sample, such as one stemming from a crowdsourcing platform, is in line with the recent body of literature. Future studies could use opt-in panel providers and quota sampling following the most recent census to obtain more representative information.

Further studies could deepen the findings on differences between taking and leaving personalities or study food foraging from an eco-tourism and gastronomy perspective. The perspectives of stakeholders, such as chefs, restaurant owners, and foraging guides, remain largely unexplored. Cross-country comparison with European countries and Australia may be of value given the impacts of COVID-19 and food price inflation. Targeting low-income populations and investigating food foraging on the background of need is of particular interest in recent times.

## Figures and Tables

**Table 1:** Sample Description.

Sample or Sub-Sample	Overall		Balancers		Takers		Leavers		U.S. Census
Frequency or Percentage	Freq	%	Freq	%	Freq	%	Freq	%	%
Age (StDev: 0.940)									
18–24	32	8.0	3	2.8	19	10.3	10	9.1	12.0
25–34	205	51.1	55	51.9	95	51.4	55	50.0	18.0
35–44	70	17.5	24	22.6	31	16.8	15	13.6	16.3
45–54	68	17.0	20	18.9	28	15.1	20	18.2	16.4
55–64	25	6.2	3	2.8	12	6.5	10	9.1	16.7
65+	1	0.2	1	0.9	0	0.0	0	0.0	20.7
Total	401	100	106	100	185	100	110	100	100
Education (StDev: 0.927)									
Did not finish high school	3	0.7	2	1.9	0	0.0	1	0.9	11.0
Finished high school	28	7.0	9	8.5	10	5.4	9	8.2	27.0
Attended university	35	8.7	10	9.4	8	4.3	17	15.5	20.0
Bachelor’s degree	247	61.6	64	60.4	115	62.2	68	61.8	29.0
Postgraduate degree	88	21.9	21	19.8	52	28.1	15	13.6	13.0
Total	401	100	106	100	185	100	110	100	100
Household Annual Income (StDev: 1.141)									
$0 to $24,999	23	5.7	7	6.6	9	4.9	7	6.4	18.0
$25,000 to $49,999	98	24.4	41	38.7	32	17.3	25	22.7	20.0
$50,000 to $74,999	165	41.1	33	31.1	79	42.7	53	48.2	18.0
$75,000 to $99,999	94	23.4	20	18.9	54	29.2	20	18.2	13.0
$100,000 or higher	21	5.2	5	4.7	11	5.9	5	4.5	31.0
Total	401	100	106	100	185	100	110	100	100
Gender (StDev: 0.501)									
Male	202	50.4	52	49.1	103	55.7	47	42.7	49.0
Female	199	49.6	54	50.9	82	44.3	63	57.3	51.0
Total	401	100	106	100	185	100	110	100	100
Region									
Northeast	105	26.2	23	21.7	53	28.6	29	26.4	17.0
South	207	51.6	58	54.7	91	49.2	58	52.7	38.0
Midwest	64	16.0	19	17.9	30	16.2	15	13.6	21.0
West	25	6.2	6	5.7	11	5.9	8	7.3	24.0
Total	401	100	106	100	185	100	110	100	100

**Table 2:** Scale Loadings, Reliabilities, and Convergent Validity for Multi-Item Scales.

Scales and Items	Takers		Balancers		Leavers		Overall					
	Mean	St Dev	Mean	St Dev	Mean	St Dev	Mean	St Dev	Loading	CRA	CR	AVE
Importance of Going Out to Experience Nature										0.753	0.859	0.670
How important is going hunting/fishing to obtain food	5.751	1.178	5.198	1.185	5.036	1.334	5.409	1.266	0.838			
How important is collecting flowers, stones, or shells	5.914	1.140	5.481	1.057	5.227	1.412	5.611	1.237	0.809			
How important is going surfing, boating, or sailing	5.703	1.136	5.292	1.149	5.282	1.484	5.479	1.261	0.808			
Importance of Tending/Harvesting Nature and Food at Home										0.758	0.838	0.510
How important is growing food and flowers in my own garden	5.903	0.913	5.575	1.081	5.491	1.249	5.703	1.075	0.741			
How important is keeping plants in my living and work environment	5.930	0.970	5.547	0.943	5.655	1.179	5.753	1.039	0.752			
How important is cooking and food preparation	5.973	0.967	5.840	1.020	5.809	0.958	5.893	0.982	0.616			
How important are food processing and preserving	5.843	1.159	5.566	1.073	5.555	1.203	5.691	1.158	0.741			
How important is keeping livestock to obtain food	5.870	1.223	5.453	1.318	5.191	1.462	5.574	1.349	0.710			
Foraging is Good for Society’s Wellbeing										0.774	0.855	0.597
Food foraging contributes to social well-being and the development of society	5.870	0.897	5.538	0.953	5.564	1.049	5.698	0.969	0.759			
Food foraging contributes to solidarity	5.865	0.911	5.302	0.953	5.582	1.073	5.638	0.997	0.727			
Food foraging contributes to distributive justice	5.741	1.023	5.330	1.114	5.455	1.233	5.554	1.122	0.755			
Food foraging is the embodiment of democracy	5.886	0.994	5.613	0.977	5.400	1.356	5.681	1.120	0.844			
Local Foragers are Knowledgeable										0.703	0.835	0.628
Where I live, collectors know where to find edible plants	5.838	0.967	5.453	1.047	5.473	1.085	5.636	1.039	0.807			
Where I live, residents appreciate the collection of edible plants	5.800	0.957	5.349	0.962	5.455	1.084	5.586	1.015	0.791			
Where I live, collectors are careful when collecting edible plants	5.897	1.011	5.613	0.896	5.545	1.101	5.726	1.021	0.779			
Foraging is Good for People and the Planet										0.641	0.805	0.580
Food foraging combines personal interests and the common good in our society	5.914	0.884	5.623	0.758	5.500	0.951	5.723	0.891	0.781			
Food foraging is ethical	5.935	1.022	5.557	0.942	5.873	0.945	5.818	0.993	0.715			
Food foraging is sustainable	5.962	0.903	5.623	0.995	5.745	1.004	5.813	0.967	0.787			
Local Foraging is Challenging										0.664	0.813	0.593
Where I live, there are few easily accessible green spaces to collect edible plants.	5.941	0.931	5.396	1.113	5.036	1.272	5.549	1.149	0.798			
Where I live, the authorities responsible for public greenspaces do not encourage collecting edible plants	5.578	1.123	5.321	1.314	5.155	1.396	5.394	1.267	0.693			
Where I live, most green spaces are too contaminated or polluted to collect edible plants	5.827	1.168	5.226	1.238	5.082	1.508	5.464	1.332	0.813			
Take/Leave Tended Patrolled Non-Garden (Cemetery/Campus)										0.799	0.908	0.831
Take all (100) vs. leave all (0) at a cemetery	82.18	14.38	64.72	15.87	42.21	26.06	66.60	24.99	0.893			
Take all (100) vs. leave all (0) at a school, institute, or other public grounds	85.05	10.48	66.46	15.92	46.96	23.09	69.69	22.74	0.931			
Take/Leave Untended Unpatrolled Non-garden (Abandoned/Roadside/Railroad)										0.910	0.936	0.787
Take all (100) vs. leave all (0) at a former farm or orchard	82.00	11.18	63.49	14.29	43.04	18.62	66.42	21.70	0.825			
Take all (100) vs. leave all (0) at an abandoned, foreclosed property	84.50	10.30	58.00	16.71	38.05	21.38	64.75	25.21	0.893			
Take all (100) vs. leave all (0) at a parking lot, empty lot, roadside	84.08	9.24	66.75	14.37	32.52	18.24	65.35	25.36	0.907			
Take all (100) vs. leave all (0) under freeways or on railroad land	83.41	10.68	59.92	14.68	34.42	19.02	63.76	25.04	0.920			
Take/Leave Tended Patrolled Garden (Botanic/Community)										0.821	0.917	0.848
Take all (100) vs. leave all (0) at a park or botanical garden	81.48	12.58	60.49	17.59	47.98	23.77	66.74	22.74	0.909			
Take all (100) vs. leave all (0) at a community garden	83.10	10.85	60.75	15.53	49.41	21.01	67.95	21.29	0.932			

Note: St Dev = Standard Deviation, CRA = Chronbach’s Alpha, CR = Composite Reliability, AVE = Average Variance Extracted.

**Table 3:** Scale Discriminant Validity.

Fornell–Larcker Criterion	A	B	C	D	E	F	G	H	I
(A) Foraging is good for people and the planet	0.762								
(B) Foraging is good for society’s wellbeing	0.568	0.773							
(C) Importance of going out to experience nature	0.335	0.568	0.818						
(D) Importance of tending/harvesting nature and food at home	0.558	0.657	0.681	0.714					
(E) Local foragers are knowledgeable	0.532	0.645	0.607	0.614	0.792				
(F) Local foraging is challenging	0.323	0.483	0.587	0.420	0.446	0.770			
(G) Take/leave tended patrolled non-garden	0.212	0.123	0.087	0.135	0.133	0.211	0.912		
(H) Take/leave tended patrolled garden	0.209	0.135	0.103	0.192	0.137	0.125	0.720	0.921	
(I) Take/leave untended, unpatrolled non-garden	0.181	0.221	0.308	0.238	0.246	0.378	0.757	0.687	0.887
Heterotrait–Monotrait Ratio	A	B	C	D	E	F	G	H	I
(b) Foraging is good for society’s wellbeing	0.800								
(C) Importance of going out to experience nature	0.472	0.741							
(D) Importance of tending/harvesting nature and food at home	0.806	0.851	0.889						
(E) Local foragers are knowledgeable	0.788	0.873	0.831	0.834					
(F) Local foraging is challenging	0.473	0.673	0.818	0.570	0.639				
(G) Take/leave tended patrolled non-garden	0.287	0.149	0.115	0.175	0.174	0.265			
(H) Take/leave tended patrolled garden	0.279	0.168	0.126	0.244	0.176	0.148	0.886		
(I) Take/leave untended, unpatrolled non-garden	0.226	0.260	0.368	0.282	0.307	0.458	0.878	0.804	

Note: Fornell–Larker criterion units—diagonal: square root of average variance extracted for scale, Others: Correlations between scales. Heterotrait–monotrait ratio units—ratio of correlations between scales.

**Table 4:** Path Coefficients for Overall Sample and Sub-samples.

Sample or Sub-Sample	Overall			Balancers			Takers			Leavers		
Path Coefficient, T Statistic, *p* Value	Coef	T Stat	*p* Val	Coef	T Stat	*p* Val	Coef	T Stat	*p* Val	Coef	Tstat	*p* Val
Hypothesized Relationship												
H1a: Importance of going out to experience nature → foraging is good for society’s wellbeing	**0.225**	2.702	0.007	**0.414**	3.951	0.000	0.142	1.283	0.199	0.170	0.956	0.339
H1c: Importance of going out to experience nature → local foragers are knowledgeable	**0.352**	5.034	0.000	0.208	1.775	0.076	**0.373**	3.328	0.001	**0.426**	3.285	0.001
H1b: Importance of going out to experience nature → foraging is good for people and planet	−0.084	1.132	0.258	−0.115	0.986	0.324	0.026	0.206	0.837	**−0.296**	2.071	0.038
H1d: Importance of going out to experience nature → local foraging is challenging	**0.561**	8.369	0.000	**0.488**	4.221	0.000	**0.627**	6.190	0.000	**0.514**	3.509	0.000
H2a: Importance of tending/harvesting nature and food at home → foraging is good for society’s wellbeing	**0.504**	6.164	0.000	**0.320**	3.039	0.002	**0.681**	8.439	0.000	**0.446**	2.373	0.018
H2c: Importance of tending/harvesting nature and food at home → local foragers are knowledgeable	**0.374**	5.054	0.000	**0.477**	4.373	0.000	**0.418**	3.876	0.000	0.244	1.646	0.100
H2b: Importance of tending/harvesting nature and food at home → foraging is good for people and the planet	**0.615**	9.070	0.000	**0.552**	5.092	0.000	**0.613**	5.557	0.000	**0.722**	5.560	0.000
H2d: Importance of tending/harvesting nature and food at home → local foraging is challenging	0.038	0.436	0.663	0.172	1.010	0.312	0.153	1.294	0.196	−0.214	1.309	0.191
H3a: Foraging is good for society’s wellbeing → take/leave tended patrolled non-garden	−0.075	0.852	0.394	0.102	0.518	0.604	0.019	0.147	0.883	−0.201	1.256	0.209
H3b: Foraging is good for society’s well-being → take/leave untended unpatrolled non-garden	−0.019	0.227	0.821	0.133	0.607	0.544	0.030	0.248	0.804	0.009	0.067	0.947
H3c: Foraging is good for society’s well-being → take/leave tended patrolled garden	−0.014	0.157	0.876	−0.014	0.070	0.944	−0.042	0.402	0.688	−0.056	0.312	0.755
H5a: Local foragers are knowledgeable → take/leave tended patrolled non-garden	−0.008	0.101	0.919	−0.110	0.783	0.433	0.077	0.537	0.592	−0.246	1.576	0.115
H5b: Local foragers are knowledgeable → take/leave untended unpatrolled non-garden	0.089	1.065	0.287	−0.275	1.181	0.238	0.016	0.113	0.910	0.198	1.762	0.078
H5c: Local foragers are knowledgeable → take/leave tended patrolled garden	0.018	0.194	0.846	−0.044	0.215	0.830	−0.038	0.352	0.725	−0.103	0.401	0.689
H4a: Foraging is good for people and planet → take/leave tended patrolled non-garden	**0.199**	2.692	0.007	**0.336**	2.267	0.023	**0.305**	2.579	0.010	0.239	1.816	0.069
H4b: Foraging is good for people, and planet → take/leave untended unpatrolled non-garden	0.036	0.504	0.614	0.352	1.374	0.170	**0.384**	2.991	0.003	**−0.298**	2.636	0.008
H4c: Foraging is good for people and planet → take/leave tended patrolled garden	**0.187**	2.619	0.009	−0.079	0.463	0.643	**0.464**	4.801	0.000	0.192	1.578	0.115
H6a: Local foraging is challenging → take/leave tended patrolled non-garden	**0.187**	2.240	0.025	**−0.315**	2.203	0.028	−0.048	0.399	0.690	−0.032	0.189	0.850
H6b: Local foraging is challenging → take/leave untended unpatrolled non-garden	**0.335**	5.093	0.000	−0.050	0.209	0.834	−0.064	0.474	0.636	**0.321**	3.052	0.002
H6c: Local foraging is challenging → take/leave tended patrolled garden	0.063	0.789	0.430	−0.118	0.505	0.614	−0.097	0.857	0.391	−0.319	2.120	0.034

Bold = *p* < 0.05, green = significant overall and in sub-group, yellow = significant overall but not in sub-group, red = not significant overall but significant in sub-group.

## Data Availability

The data presented in this study are available on request from the corresponding author.
